# Transcriptomic, Redox Status and Adipocytokine Profiles in Metabolic Dysfunction-Associated Steatotic Liver Disease: Impact of Coexisting Type 2 Diabetes

**DOI:** 10.3390/medsci13040326

**Published:** 2025-12-18

**Authors:** Sanja Erceg, Ana Ninić, Jelena Kotur-Stevuljević, Omar Ben Mariem, Miloš Mitrović, Jelena Munjas, Miron Sopić, Boško Misita, Milica Mamić, Aleksandra Klisic, Ratko Tomašević

**Affiliations:** 1 Department of Medical Biochemistry, Faculty of Pharmacy, University of Belgrade, 11000 Belgrade, Serbia; sanja.erceg@pharmacy.bg.ac.rs (S.E.); jelena.kotur@pharmacy.bg.ac.rs (J.K.-S.); jelenaj@pharmacy.bg.ac.rs (J.M.); miron.sopic@pharmacy.bg.ac.rs (M.S.); misitabosko@yahoo.com (B.M.); 2 Department of Pharmacological and Biomolecular Sciences, University of Milan, 20133 Milan, Italy; omar.benmariem@unimi.it; 3 Clinical Department for Gastroenterology and Hepatology, University Medical Center Zvezdara, 11120 Belgrade, Serbia; dr.milosh.mitrovic@gmail.com; 4 Department of Laboratory Diagnostics, Clinical Hospital Center Zemun, 11080 Belgrade, Serbia; mamicmilics8@gmail.com; 5 Faculty of Medicine, University of Montenegro, 81000 Podgorica, Montenegro; aleksandranklisic@gmail.com; 6 Center for Laboratory Diagnostics, Primary Health Care Center, 81000 Podgorica, Montenegro; 7 Faculty of Medicine, University of Belgrade, 11000 Belgrade, Serbia; tomasevicratko@gmail.com; 8 Department of Gastroenterology and Hepatology, Clinic for Internal Medicine, Clinical Hospital Center Zemun, 11000 Belgrade, Serbia

**Keywords:** MASLD, T2D, AOPP, PON1, CD36, TLR9, GPX1, adiponectin, leptin, resistin

## Abstract

Background: Metabolic dysfunction-associated steatotic liver disease (MASLD) commonly coexists with type 2 diabetes (T2D), but their independent contributions to redox imbalance, inflammation and immune signaling remain uncertain. Objectives: This study aimed to evaluate whether the presence of MASLD alone, and the presence of T2D within MASLD, are independently associated with high-risk profiles of oxidative/antioxidant markers, peripheral blood mononuclear cell (PBMC) gene expression and adipocytokines. Methods: A total of 190 participants were categorized via abdominal ultrasound as controls (n = 46), MASLD (n = 83) or MASLD with T2D (n = 61). Measurements included advanced oxidation protein products (AOPP) and paraoxonase-1 (PON1) activity in serum; messenger ribonucleic acids expression of cluster of differentiation 36 (CD36), Toll-like receptor 9 (TLR9), and glutathione peroxidase-1 in PBMC; and adiponectin, leptin, and resistin in plasma. Biomarker values were adjusted and statistical comparisons among groups were performed using the Quade test. Subsequently, biomarkers were stratified into tertiles to examine associations between high-risk biomarker levels and the presence of MASLD or T2D in patients with MASLD using multivariate binary logistic regression. Results: Multivariate analysis showed that MASLD presence was independently associated with both increased AOPP and decreased resistin levels in the circulation. Furthermore, T2D presence in patients with MASLD was independently associated with increased CD36 and decreased TLR9 gene expression in PBMCs, as well as elevated circulating leptin levels. Conclusions: Collectively, these findings underscore the complex interplay between oxidative stress, insulin resistance, inflammation, and immune signaling in the pathogenesis of MASLD, which are fundamental factors contributing to this condition.

## 1. Introduction

Metabolic dysfunction-associated steatotic liver disease (MASLD) is currently the most common chronic liver disease worldwide, affecting more than one third of adults. It is closely related to obesity and type 2 diabetes (T2D) [1]. Up to 70% of T2D patients also have MASLD [2]. Both diseases share common features, including dyslipidemia, insulin resistance (IR), oxidative stress (OS), and inflammation [3]. Their interaction is synergistic, accelerates disease progression and increases the risk of hepatic and extrahepatic complications [4]. MASLD refers to a range of histological changes that begin with steatosis and may progress to metabolic dysfunction-associated steatohepatitis (MASH), which includes steatosis as well as varying degrees of hepatic inflammation and fibrosis [1].

Although the pathogenesis of MASLD is not yet fully understood, it is assumed that several simultaneous and interrelated factors are involved, which Buzzetti and colleagues [5] termed the “multiple-hit hypothesis”. OS is one such contributing factor. Hepatic steatosis leads to mitochondrial and peroxisomal dysfunction, disrupting cellular respiration and resulting in the accumulation of toxic lipid intermediates. This cascade increases the production of reactive oxygen species (ROS) beyond the capacity of the antioxidant defense system, ultimately leading to OS [5]. To capture both oxidative damage and endogenous defense, we selected two complementary plasma markers. Advanced oxidation protein products (AOPP) are markers of protein oxidation formed when prooxidants modify plasma proteins such as albumin and fibrinogen, leading to the formation of dityrosine-containing cross-linked protein aggregates [6]. AOPP were first identified in the plasma of patients with chronic uraemia in 1996 [7] and have since been associated with chronic inflammation and metabolic diseases, including liver diseases. AOPP promote inflammation by inducing proinflammatory cytokines, while their contribution to fibrosis occurs through epithelial-to-mesenchymal transition (EMT) in hepatocytes, implicating them in MASLD pathogenesis [6,8]. Conversely, paraoxonase 1 (PON1) is a liver-derived enzyme with antioxidant and anti-inflammatory properties. Circulating bound to high-density lipoprotein (HDL), PON1 hydrolyses lipid peroxides in both HDL and low-density lipoprotein (LDL), thereby preserving HDL functionality and preventing oxidative modification of LDL [9]. By measuring AOPP (damage) together with PON1 (defense), we aimed to examine both sides of the redox imbalance that contributes to MASLD development and progression.

IR increases lipolysis in adipose tissue, thereby enhancing the flow of fatty acids to the liver. This is only one aspect of the complex interplay between the liver and adipose tissue in MASLD. Additionally, IR contributes to adipose tissue dysfunction, leading to altered production and secretion of adipocytokines [10]. This dysregulation is characterized by increased levels of proinflammatory mediators such as leptin and resistin, and reduced levels of anti-inflammatory mediators such as adiponectin. Although leptin contributes to metabolic homeostasis at physiological levels, increased leptin concentrations promote OS and activate Kupffer cells. In contrast, adiponectin, the most abundant adipocytokine in the human body, improves insulin sensitivity, reduces OS, inhibits hepatocyte apoptosis, and has anti-inflammatory effects [10]. Resistin is a proinflammatory adipocytokine which, unlike leptin and adiponectin, is secreted by macrophages that infiltrate adipose tissue rather than by adipocytes themselves [11]. It contributes to metabolic dysfunction by promoting lipid accumulation, increasing endoplasmic reticulum stress, and stimulating inflammatory processes [10].

Both MASLD and T2D are characterized by chronic, low-grade inflammation driven by the release of proinflammatory mediators from various cell types [10]. Among these, peripheral blood mononuclear cells (PBMCs), which include lymphocytes and monocytes, play a key role as circulating immune cells capable of recognizing and responding to systemic inflammatory signals. Consequently, PBMC transcriptomics can provide a blood-based signature of systemic immune activity and inflammation-related changes [12]. To address this, we focused on three PBMC genes that reflect key aspects of MASLD pathogenesis: Lipid uptake, immune activation, and antioxidant defense. Cluster of differentiation 36 (CD36) is a scavenger receptor involved in the uptake of long-chain fatty acids and oxidized (ox)LDL, thereby linking lipid accumulation with inflammation in immune cells [13]. Toll-like receptor 9 (TLR9) is expressed in the endoplasmic reticulum and, upon stimulation with CpG motifs present in both microbial and mitochondrial (mt)DNA, is recruited to endosomal compartments. In MASLD, mtDNA is particularly relevant, as it can be released from hepatocytes and immune cells under lipid overload and OS-induced injury. Activation of TLR9 by mtDNA further stimulates NF-κB signaling and proinflammatory cytokine production, thereby linking cellular stress to the chronic low-grade inflammation typical of MASLD and T2D [14]. Glutathione peroxidase 1 (GPX1) is an antioxidant enzyme that reduces hydrogen peroxide and lipid peroxides, limiting ROS accumulation and preserving redox balance. In MASLD, where excessive lipid accumulation contributes to OS, compromised GPX1 activity may lead to increased lipid peroxidation, mitochondrial dysfunction, and enhanced inflammatory signaling [15]. PBMCs are frequently used for gene expression analyses as they are accessible through simple, non-invasive and inexpensive blood sampling [12].

Given the growing recognition of the complex interplay between metabolic dysfunction, OS, immune signaling, and inflammation in MASLD, particularly with comorbid T2D, this study was designed to investigate circulating biomarkers that may reflect these underlying mechanisms. Building on our previous findings highlighting the significant impact of T2D on the clinical and metabolic profile of MASLD patients [16], we applied a similar approach by categorizing patients into MASLD and MASLD + T2D subgroups. The aim was to determine whether MASLD and/or T2D within MASLD are independently associated with high-risk levels of gene expression, redox status markers, and adipocytokines.

## 2. Materials and Methods

### 2.1. Participants

A total of 190 study participants were consecutively recruited from individuals attending routine gastroenterology outpatient appointments that underwent abdominal ultrasound at the Clinical Hospital Center “Zemun” and the University Medical Center “Zvezdara” from January 2020 to March 2023. They were divided into three groups: MASLD + T2D (61), MASLD (83) and control group (CG) (46). T2D diagnosis was conducted in accordance with the American Diabetes Association guideline [17] and the Serbian national guideline [18]. All participants gave informed consent and the study was ethically approved by the relevant committees of the University of Belgrade—Faculty of Pharmacy (Protocol No. 835/2; date of approval: 11 April 2022), of the Clinical Hospital Center Zemun (Protocol No. 733/1; date of approval: 17 October 2019) and of the University Medical Center Zvezdara (Protocol No. 3/2022/1, date of approval: 18 March 2022). The study was conducted in accordance with the ethical principles of the Declaration of Helsinki [19]. Participants completed a questionnaire collecting demographic, anthropometric, and clinical information such as age, sex, weight, height, waist circumference (WC), systolic and diastolic blood pressure (SBP and DBP, respectively), comorbidities, family history, medications, smoking, alcohol use, and exercise habits. Body mass index (BMI) was calculated as weight (kg) divided by squared height (m^2^).

Individuals with secondary causes of liver injury were excluded, including viral or autoimmune hepatitis, biliary diseases, cirrhosis, current or prior alcohol abuse, liver transplantation, hepatocellular carcinoma or other cancers, and kidney disease. Additionally, individuals with T1D were excluded because it represents a distinct metabolic phenotype from T2D. Moreover, genetic conditions such as Wilson’s disease and α1-antitrypsin deficiency were also excluded because they are secondary causes of steatosis. Individuals with acute-phase diseases were excluded. Individuals with symptoms or clinical evidence of acute infection, acute inflammatory illness, recent surgery, trauma, or hospitalization were not eligible, ensuring that only clinically stable adults were enrolled. Additionally, none of the study participants had thyroid disorders. All exclusion criteria listed above were determined by clinical evaluation and review of anamnesis, laboratory findings, and medical records conducted by gastroenterologists and hepatologists at the participating medical centers, according to standard diagnostic criteria.

### 2.2. Blood Biochemistry

Fasting serum and whole blood samples in K_2_EDTA tubes were collected from participants at the Clinical Hospital Center “Zemun” and the University Medical Center “Zvezdara.” Blood was centrifuged (1500 rcf, 10 min) within one hour of collection. Serum was analyzed for lipid profile (total cholesterol (TC), triglycerides (TG), HDL-C) and glucose by spectrophotometric methods on DxC 700 AU and DxC 480 AU analyzers (Beckman Coulter, Brea, CA, USA). LDL-C was calculated using the Friedewald formula for participants with TG ≤ 4.52 mmol/L [20], while for those with TG > 4.52 mmol/L a direct LDL-C assay was used on the same analyzer platforms. C-reactive protein (CRP) was measured by immunoturbidimetry, while the activities of alanine aminotransferase (ALT), aspartate aminotransferase (AST), alkaline phosphatase (ALP) and γ-glutamyl transferase (GGT) were assessed with enzymatic assays on the same analyzer platforms. HbA1c in whole blood was also determined on the same analyzer platforms using an immunoturbidimetric method. Serum and whole blood vacutainers not immediately processed were quickly transported to the University of Belgrade—Faculty of Pharmacy, underwent centrifugation under the same conditions (1500 rcf, 10 min), and aliquots of serum and plasma were stored at −80 °C for further analysis.

### 2.3. Redox Status Markers

The activity of PON1 was measured by hydrolysis of paraoxon and diazoxon with quantification of p-nitrophenol at 412 nm and expressed in U/L [21]. For PON1 the intra- and inter-assay coefficients of variation (CV) were 3.6% and 9.3%, respectively. AOPP were determined at 340 nm with glacial acetic acid and potassium iodide and expressed as μmol/L chloramine T equivalents [7]. For AOPP, the intra- and inter-assay CV were 1.15% and 4.09%, respectively. The ILAB 300 Plus analyzer (Instrumentation Laboratory, Milan, Italy) was used for these spectrophotometric analyses in serum. These assays are routinely performed in our laboratory and have been previously validated for clinical and research use.

### 2.4. Ribonucleic Acid (RNA) Isolation from PBMCs and Quantification of Messenger (m)RNAs

Total RNA was isolated from PBMCs using organic extraction with TRIzol™ reagent (Invitrogen, Waltham, MA, USA). The procedure was optimized according to laboratory protocols established by the Department of Medical Biochemistry [22]. RNA concentration was measured at 260 nm, and its purity was assessed by A260/A280 and A260/A230 ratios. All RNA samples were stored at −80 °C prior to reverse transcription. Complementary (c) DNA was synthesized with the High-Capacity cDNA Reverse Transcription Kit (Applied Biosystems, Waltham, MA, USA) following the manufacturer’s instructions. Quantitative mRNA analysis for TLR9, GPX1, CD36 and glyceraldehyde-3-phosphate dehydrogenase (GAPDH) was performed using HOT FIREPol^®^ EvaGreen^®^ qPCR Mix Plus (Solis BioDyne, Tartu, Estonia) and gene-specific primers on a 7500 Real-Time PCR System (Applied Biosystems, Waltham, MA, USA). Primer sequences (5′→3′) are provided in Supplementary Table S1. GAPDH served as the reference (endogenous) gene for normalization. Data analysis was performed using SDS Software v1.4.0.25 (Applied Biosystems, Waltham, MA, USA). Quantification used the standard-curve method with serial dilutions of pooled cDNA. Across plates, standard-curve slopes ranged from −3.1 to −3.6 (≈110–90% efficiency), and all calibrations showed R^2^ ≥ 0.99. Sample Cq values were converted to mRNA levels using the corresponding curve and normalized to GAPDH: Normalized expression (target) = target mRNA level/GAPDH mRNA level

### 2.5. Adipocytokines

Resistin, adiponectin, and leptin in plasma were quantified using commercial ELISA DuoSet kits (DY1359, DY1065, DY398; R&D Systems, Minneapolis, MN, USA) and SPECTROstar Nano (BMG Labtech, Ortenberg, Germany). Detection ranges were 31.2–2000 pg/mL for both resistin and leptin and 62.5–4000 pg/mL for adiponectin. Plasma was diluted 1:80 (resistin), 1:30,000 (adiponectin), and 1:70 (leptin). Assays followed the manufacturer’s protocols. The adiponectin-leptin (AL) ratio was calculated by dividing adiponectin level by leptin level.

### 2.6. Statistical Analysis

The Shapiro–Wilk test and the Kolmogorov–Smirnov test were employed to assess the normality of data distributions. All parameters except one (WC) did not meet the criteria for normal distribution (*p* < 0.05). As a result, the data are reported as median values with interquartile ranges (Q1–Q3) for consistency. Categorical variables are presented as both absolute and relative frequencies.

mRNA levels, redox status markers and adipocytokines with the AL ratio were adjusted for age, antihypertensives (AHT) and/or cardiovascular disease therapy (CVD Tx), and exercise using the Quade test (non-parametric ANCOVA), to assess whether these factors influenced the differences in biomarker levels between groups. To avoid overfitting and multicollinearity, covariates were inspected for associations using chi-square tests and Spearman correlation analysis. AHT and/or CVD Tx was significantly associated with lipid-lowering therapy (χ^2^ = 14.95, *p* < 0.001; Phi = 0.281) and with the presence of hypertension (HTN) and/or CVD (χ^2^ = 55.56, *p* < 0.001; Phi = 0.541), while WC strongly correlated with BMI (ρ = 0.760, *p* < 0.001). Consequently, BMI and WC were not included in the final models, as they are closely linked to metabolic dysfunction and represent a core component of the MASLD phenotype. Insulin and oral antidiabetic drugs (OAD) Tx were not included because they were specific to the MASLD + T2D group.

Because adiponectin, leptin and the AL ratio are known to exhibit sex-related differences, these markers were analyzed separately in males and females. As male participants differed across groups with respect to age, HTN and/or CVD presence and exercise, analyses in males were adjusted for these covariates. In females, group differences were adjusted for age, HTN and/or CVD presence, and alcohol consumption.

Group differences in continuous markers were assessed using the Kruskal–Wallis test, followed by post hoc analysis using the Mann–Whitney U test. Group differences in adjusted biomarkers were assessed using the Quade test, with the Games–Howell test applied post hoc. Categorical variables were analyzed using the chi-square test for contingency tables.

Redox status markers, mRNA expression, and adipocytokines were divided into tertiles. High-risk tertiles were coded as 1, while the other two tertiles were coded as 0. For PON1, TLR9 mRNA, GPX1 mRNA, adiponectin, and resistin, the lowest tertile was classified as high risk; conversely, for AOPP, CD36 mRNA, and leptin, the highest tertile represented high risk. These risk classifications were determined based on group-specific patterns observed in this study. Tertiles were applied due to the absence of established clinical cut-offs or reference ranges for the analyzed biomarkers. As such, the tertile-based findings are hypothesis-generating and should be interpreted with caution. Univariate binary logistic regression analysis was subsequently performed to examine associations between the presence of MASLD or T2D within MASLD and high-risk tertiles of these biomarkers.

High-risk biomarker tertiles which were significantly associated with MASLD or T2D within MASLD in univariate binary logistic regression analysis were included in multivariate binary logistic regression analysis. In addition to the presence of MASLD or T2D within MASLD, the biomarkers included in the models were continuous variables that showed significant correlations with outcomes in Spearman correlation analysis. Due to the relatively large number of significant correlations, Spearman’s correlation analysis was also applied to assess intercorrelations among potential covariates. Variables showing significant mutual correlations were not included simultaneously in the final models to avoid multicollinearity. Furthermore, considering the available sample size, no final model included more than six variables. This approach follows the recommendations of Peduzzi et al. [23] for the minimum number of events per variable in logistic regression to ensure stable and unbiased estimates. Stepwise forward likelihood ratio (LR) was used for biomarker selection.

Results with *p*-values below 0.05 were considered significant.

## 3. Results

The characteristics of the studied populations are presented in Table 1. Patients with MASLD + T2D were significantly older compared to those with MASLD and the CG. Both patient groups had significantly higher BMI and WC compared to the CG, with the highest BMI observed in the MASLD + T2D group. The prevalence of HTN and/or CVD was significantly higher in both patient groups compared to the CG, as was the use of AHT and/or CVD Tx. Participants in the CG were more physically active compared to MASLD patients. As expected, antidiabetic therapy (insulin and/or OAD Tx) was present exclusively in the MASLD + T2D group. The use of lipid-lowering Tx was significantly higher in MASLD + T2D compared to the CG.

Table 2 summarizes the biochemical markers, redox status markers, mRNAs expression and adipocytokines in all participants. Glucose, HbA1c, TG and CRP were highest in MASLD + T2D patients. In contrast, HDL-C was lowest in MASLD + T2D patients. LDL-C was significantly higher in MASLD patients compared to MASLD + T2D patients. Liver enzymes (ALT, ALP and GGT) were significantly higher in both patient groups compared to the CG. Although PON1 activity did not differ significantly between the three groups (borderline significance), MASLD + T2D patients exhibited significantly lower PON1 activity compared to the CG. AOPP levels were significantly elevated in both patient groups compared to the CG. TLR9 mRNA expression was significantly lower, while CD36 mRNA expression was significantly higher in MASLD + T2D patients compared to both MASLD patients and the CG. GPX1 mRNA expression was significantly reduced in both patient groups compared to the CG. Among the adipocytokines, only adiponectin showed a significant difference between groups, with the lowest levels observed in the MASLD + T2D patients. Resistin and leptin levels did not differ significantly between groups.

To investigate the association between biomarker high-risk tertiles and the presence of MASLD, univariate binary logistic regression analyses were performed (Table 3). MASLD presence was associated with 2.5-fold and 3.3-fold increases in the odds of belonging to the high-risk tertiles of PON1 and AOPP, respectively. Furthermore, MASLD was associated with higher odds of GPX1 mRNA (OR = 3.1), CD36 mRNA (OR = 2.5), and resistin (OR = 2.7) being in the high-risk tertile.

Stepwise multivariate binary logistic regression analyses were performed to determine whether MASLD presence was significantly and independently associated with markers being in the high-risk tertiles (Table 4). Only markers whose high-risk tertiles (coded markers) were significantly associated with MASLD presence in univariate binary logistic regression analysis were included as outcomes in the multivariate models. Potential covariates for adjustment were selected based on Supplementary Table S2, which indicates correlations between the outcomes (in their continuous form) and other continuous variables. MASLD presence was independently associated with high-risk tertiles of resistin and AOPP, with approximately 2.5-fold and 5-fold higher odds, respectively.

To explore the association between biomarker high-risk tertiles and the presence of T2D in MASLD, univariate binary logistic regression analyses were conducted (Table 5). The presence of T2D in MASLD was associated with a 2.9-fold increase in the odds of AOPP levels, a 3.7-fold increase in the odds of TLR9 mRNA expression, and a 2.1-fold increase in the odds of CD36 mRNA expression being in the high-risk tertile. In addition, T2D in MASLD was associated with a 3.0-fold increase in the odds of leptin levels belonging to the high-risk tertile.

Stepwise multivariate binary logistic regression analyses were performed to determine whether the presence of T2D in MASLD was independently associated with markers being in the high-risk tertiles (Table 6). Only those markers whose high-risk tertiles (coded markers) were significantly associated with the presence of T2D in MASLD in univariate binary logistic regression analysis were included as outcomes in the multivariate models. Potential covariates for adjustment were selected based on Supplementary Table S2, which indicates correlations between the outcomes (in their continuous form) and other continuous variables. The presence of T2D in MASLD was independently associated with high-risk tertiles of CD36 mRNA expression, TLR9 mRNA expression, and leptin levels, with approximately 4.2-fold, 3.9-fold, and 2.5-fold higher odds, respectively.

## 4. Discussion

We examined biomarkers of dyslipidemia, IR, OS, and inflammation in MASLD patients with and without T2D, to assess whether MASLD itself and the presence of T2D within MASLD are independently associated with high-risk levels of gene expression, redox status markers and adipocytokines. Since established clinical cut-offs for the selected markers are not available, tertile-based stratification was used as an exploratory approach, and the findings should be considered hypothesis-generating. Observed associations may reflect disease consequences rather than pathogenic mechanisms.

### 4.1. AOPP and Resistin in Relation to MASLD

Our results indicate that oxidative protein damage plays a significant role in MASLD, as demonstrated by the independent association between MASLD presence and increased AOPP (Table 4). AOPP are markers of oxidative modification of plasma proteins, primarily albumin and fibrinogen, which are synthesized in the liver [6,24]. Therefore, increased hepatic OS directly contributes to higher circulating AOPP. This process is accompanied by reduced antioxidant capacity, making plasma proteins more susceptible to oxidative modification [24,25]. Impaired antioxidant defense is supported by lowered PON1 activity in MASLD + T2D patients, as well as reduced GPX1 gene expression in both patient groups compared with the CG (Table 2). Although the overall group difference for PON1 did not reach statistical significance, post hoc analysis revealed significantly lower PON1 activity in the MASLD + T2D group compared to the CG (Table 2).

AOPP also significantly differed between patient groups and CG (Table 2). Several studies, including ours, have found higher AOPP in MASLD patients than in healthy controls [26,27,28]. Notably, two studies focused solely on MASH cases [27,28]. Although the stages of MASLD were not specified in our patient groups, both demonstrated greater inflammation than the CG, as illustrated by CRP levels in Table 2. CRP levels were highest in the MASLD + T2D group, emphasizing the presence of chronic low-grade inflammation in both MASLD and T2D [29]. Previous studies have shown that AOPP can bind to the CD36 receptor, particularly in renal tubular cells, and initiate inflammatory signaling via NADPH oxidase activation [30,31]. Therefore, AOPP not only reflect plasma protein oxidation but also act as inflammatory mediators, potentially linking hepatic OS to inflammation that drives MASLD progression [28,32].

Besides elevated AOPP, MASLD presence was independently associated with decreased plasma resistin (Table 4). However, resistin levels did not significantly differ among the studied groups (Table 2). A recent meta-analysis [33] reported generally higher circulating resistin levels in MASLD compared with controls, but lower resistin levels in biopsy-confirmed MASH cases, suggesting a decrease in resistin with increasing disease severity and possible progression of fibrosis. This supports the concept that resistin regulation may differ between early and advanced stages of MASLD. Furthermore, while resistin has been shown to promote IR and T2D in experimental mouse models, human studies have not consistently confirmed this relationship [34,35], which may partly explain the lack of difference between the MASLD + T2D group and the CG in our study (Table 2). Unlike adiponectin and leptin [36], resistin was identified later [37] and, although often described as an adipokine, in humans it is mainly produced by immune cells, especially monocytes and macrophages [11,38]. This suggests that resistin is more closely related to immune activation and inflammatory processes than to adipose tissue mass itself. In addition, resistin may circulate in different molecular forms in human blood, which could partly explain variability in its measurement and differences between studies using different assay systems [38]. Since resistin primarily originates from inflammatory cells, its circulating levels may also reflect changes in immune cell activity during chronic low-grade inflammation, rather than only metabolic status.

### 4.2. CD36 mRNA, TLR9 mRNA and Leptin in Relation to T2D in MASLD

We analyzed the expression of several genes in PBMCs, including CD36, a multifunctional transmembrane receptor that primarily binds long-chain fatty acids and oxLDL and is expressed on various cell types, including PBMCs [13]. Its increased expression in MASLD + T2D patients suggests increased lipid uptake by immune cells in the setting of chronic hyperglycemia (Table 2). Additionally, the presence of T2D in MASLD was independently associated with elevated CD36 mRNA levels (Table 6). Supporting our findings, Sampson et al. [39] showed that CD36 expression was already elevated in monocytes of T2D patients under basal conditions, and that acute hyperglycemia did not further increase its expression, indicating long-term metabolic adaptation and possible saturation of regulatory mechanisms [39]. Additionally, hepatic CD36 mRNA levels positively correlated with steatosis severity in obese insulin-resistant rats, emphasizing its role in hepatic lipid accumulation [40].

Among its various roles, CD36 also belongs to the family of PRRs, named after their ability to recognize conserved structural motifs in biological molecules [41]. Another member of the PRR family is TLR9, which is involved in innate immune sensing [42]. PRRs recognize various ligands, including unmethylated CpG motifs, which are characteristic of microbial and mtDNA. In particular, mtDNA released from damaged mitochondria can activate TLR9, thereby linking cellular injury to immune activation [14,43]. While CD36 is mainly found on the cell surface, TLR9 is located intracellularly, which allows it to detect released mtDNA. In the liver, activation of TLR9 in Kupffer cells induces the production of proinflammatory cytokines such as transforming growth factor β (TGF-β), interleukin 1β (IL-1β) and tumor necrosis factor α (TNFα), which promote hepatic stellate cell (HSC) activation, hepatocyte lipid accumulation, and apoptosis. Thus, TLR9 signaling links not only cellular injury to immune activation, but also to fibrogenesis [42].

Despite this well-established proinflammatory role, chronic metabolic stress and persistent low-grade inflammation may lead to sustained TLR9 stimulation and the development of TLR tolerance [44]. This adaptive mechanism is characterized by reduced transcription of inflammatory mediators following prolonged receptor activation. TLR tolerance has been described primarily in monocytes, macrophages, and dendritic cells and is thought to serve as a protective response against excessive inflammatory injury. Although TLR tolerance arises from alterations within the signaling pathway, it may also involve a reduction in receptor abundance. One proposed mechanism is that the toll-interleukin 1 receptor (TIR) domain of TLR9 can recruit ubiquitin-protein ligases, such as Triad3A, which tag the receptor for degradation [45]. The study by Alegre et al. [46] demonstrated significantly lower TLR9 mRNA expression in both PBMCs and liver lymphocytes from patients with simple steatosis compared to controls and proposed enhanced ubiquitin-dependent regulation as a possible mechanism. This could also explain the lower TLR9 mRNA levels observed in MASLD + T2D patients in our study (Table 2), as well as the independent association between T2D in MASLD and reduced TLR9 gene expression (Table 6). Notably, Alegre et al. [46] demonstrated that reduced TLR9 mRNA expression was present not only in PBMCs but also in liver lymphocytes, supporting the relevance of circulating immune cell findings for hepatic immune regulation. However, since TLR9 expression in our study was assessed exclusively in PBMCs, direct conclusions regarding its regulation within hepatic immune cells, such as Kupffer cells, should still be interpreted with appropriate caution.

The presence of T2D within MASLD was also identified as being independently associated with increased plasma leptin levels (Table 6). A 2015 meta-analysis [47] reported higher circulating leptin levels in MASLD patients compared to controls, while a 2024 meta-analysis by Makri and colleagues [48] found no difference in circulating leptin levels between MASLD patients without fibrosis and controls, but significantly higher levels in those with fibrosis. This points to a closer link between leptin and fibrosis than with MASLD alone. This may explain why leptin levels did not differ significantly between the study groups in our cohort (Table 2). Leptin’s profibrogenic effects include increasing tissue inhibitor of metalloproteinase-1 and cytokine production in activated HSCs, also upregulating TGF-β in Kupffer cells [47]. It is also important to note that leptin plays a dual role: At physiological levels, it has a protective effect but becomes proinflammatory and fibrotic when its regulation is disturbed [10]. Since T2D is characterized by chronic low-grade inflammation and disturbed adipocytokine balance [29], it likely accelerates fibrogenic signaling in the MASLD setting, which may account for the independent association between T2D within MASLD and elevated leptin levels observed in our study. Given that the group-wise difference in leptin was borderline, this association should be interpreted with appropriate caution.

### 4.3. Adiponectin and AL Ratio

Although adiponectin was not independently associated with MASLD or T2D within MASLD in neither univariate nor multivariate regression analyses (Table 3, Table 4, Table 5 and Table 6), it was the only adipocytokine to show a statistically significant difference between the groups, with the lowest levels observed in the MASLD + T2D group (Table 2). Our results are consistent with previous studies that found lower adiponectin levels in patients with hepatic steatosis compared to healthy individuals [49,50,51]. Its levels are decreased in MASLD due to adipose tissue dysfunction and IR, which reduce adiponectin secretion and contribute to disease progression. Moreover, Pan and colleagues [51] demonstrated that the association between hypoadiponectinemia and MASLD is particularly strong in patients with concomitant T2D. Adiponectin is considered a protective adipocytokine because it activates signaling pathways involving adenosine monophosphate-activated protein kinase (AMPK) and peroxisome proliferator-activated receptor alpha (PPARα), resulting in increased hepatic insulin sensitivity, enhanced fatty acid oxidation, and reduced lipid accumulation in the liver [10]. To further characterize adipose tissue dysfunction, we also evaluated the AL ratio, a composite marker closely linked to IR and inversely associated with low-grade chronic inflammation [52]. The significantly higher AL ratio observed in the MASLD + T2D group compared to both MASLD and the CG (Table 2) supports the presence of a more severe cardiometabolic risk profile in these patients. Finally, as leptin and adiponectin exhibit clear sex-related differences, with females generally displaying higher levels of both [53], these parameters are also presented stratified by sex in Supplementary Table S3 to allow a more comprehensive interpretation of adipocytokine regulation.

### 4.4. Limitations

Steatosis was diagnosed by abdominal ultrasound without histological confirmation, which remains the gold standard for assessing liver pathology [54]. Data on the exact duration of MASLD and T2D, as well as the presence and severity of T2D-related complications, were incomplete and could not be included in the analyses. The cross-sectional design of the study limits causal interpretation and the assessment of temporal changes in the examined biomarkers; therefore, we used the wording “independently associated with” rather than predictive terminology throughout the manuscript. Due to the exploratory nature of the study and the moderate sample size, no formal correction for multiple testing was applied, which may increase the risk of type I error. Validation in larger, independent cohorts, preferably through longitudinal study designs, is required to confirm the robustness and clinical relevance of the observed associations and to increase statistical power.

## 5. Conclusions

This study demonstrates that MASLD presence is independently associated with both increased AOPP and decreased resistin levels in the circulation, indicating underlying OS and altered inflammatory signaling. In patients with MASLD, concomitant T2D was independently associated with increased CD36 and decreased TLR9 gene expression in PBMCs, as well as with higher circulating leptin levels. These alterations indicate a significant imbalance in the immune and metabolic processes in patients with MASLD and T2D, conditions that act synergistically. Overall, our results emphasize the complex interplay of OS, IR, inflammation and immune signaling in MASLD. Larger studies with histological staging and longitudinal follow-up design are needed to confirm clinical relevance and to determine whether these biomarkers can support diagnosis or elucidate disease mechanisms.

## Figures and Tables

**Table 1:** Baseline characteristics of study participants.

Marker	CG N = 46	MASLD N = 83	MASLD + T2D N = 61	*p*
Age, years	48 (41–62)	53 (41–62)	58 (49–66) a **, b **	0.005
Sex (males), N (%) #	13 (28.3%)	39 (47.0%)	25 (41.0%)	0.116
BMI, kg/m^2^	25.1 (22.0–27.0)	27.3 (25.4–30.5) a ***	30.3 (27.4–34.0) a ***, b **	<0.001
WC, cm	85.5 (77.0–91.5)	95.0 (85.2–104.8) a ***	96.5 (89.5–110.0) a ***	<0.001
SBP, mmHg	120 (116–130)	130 (120–140)	130 (120–140) a **	0.036
DBP, mmHg	80 (70–80)	80 (70–90)	80 (70–90)	0.100
Smoking, N (%) #	12 (26.1%)	31 (37.3%)	21 (34.4%)	0.427
Alcohol, N (%) #	17 (37.0%)	23 (27.7%)	11 (18.0%)	0.089
Exercise, N (%) #	26 (59.1%)	28 (35.0%) a *	25 (42.4%)	0.034
HTN and/or CVD, N (%) #	5 (10.9%)	40 (48.2%) a ***	22 (36.1%) a **	0.001
Insulin Tx, N (%) #	0 (0.0%)	0 (0.0%)	23 (37.7%) a ***, b ***	<0.001
OAD Tx, N (%) #	0 (0.0%)	0 (0.0%)	48 (78.7%) a ***, b ***	<0.001
Lipid-lowering Tx, N (%) #	2 (4.3%)	9 (10.8%)	14 (23.0%) a **	0.013
AHT and/or CVD Tx, N (%) #	11 (23.9%)	43 (51.8%) a **	30 (49.2%) a **	0.006

**Abbreviations:** CG, control group; MASLD, metabolic dysfunction-associated steatotic liver disease; T2D, type 2 diabetes; BMI, body mass index; WC, waist circumference; SBP, systolic blood pressure; DBP, diastolic blood pressure; HTN, hypertension; CVD, cardiovascular disease; OAD, oral antidiabetic drugs; Tx, therapy; AHT, antihypertensives. **Data presentation:** Data are presented as median (Q1–Q3) for continuous variables and as counts (N, %) for categorical variables. Group differences were tested using the Kruskal–Wallis test and Chi-square test (#). Post hoc comparisons: * *p* < 0.05, ** *p* < 0.01, *** *p* < 0.001 (Mann–Whitney U for continuous, Chi-square for categorical). Group comparisons: a—MASLD and MASLD + T2D vs. CG; b—MASLD + T2D vs. MASLD.

**Table 2:** Biochemical markers, redox status markers and mRNA expression levels of study participants.

Marker	CG N = 46	MASLD N = 83	MASLD + T2D N = 61	*p*
Glucose, mmol/L	5.1 (4.8–5.4) a	5.4 (4.9–5.8) a **	7.5 (6.1–9.7) a ***, b ***	<0.001
HbA1c, %	5.2 (4.9–5.4)	5.4 (5.2–5.6) a ***	8.0 (6.7–9.9) a ***, b ***	<0.001
TC, mmol/L	5.28 (4.62–5.92)	5.28 (4.65–6.10)	4.86 (3.98–5.78)	0.101
TG, mmol/L	0.88 (0.70–1.41)	1.30 (0.97–1.61) a **	1.92 (1.45–2.90) a ***, b ***	<0.001
HDL-C, mmol/L	1.72 (1.41–1.92)	1.38 (1.16–1.74) a **	1.20 (0.86–1.40) a ***, b ***	<0.001
LDL-C, mmol/L	3.10 (2.48–3.90)	3.30 (2.70–4.20)	2.94 (1.85–3.70) b **	0.046
ALT, U/L	20 (16–25)	28 (18–39) a **	26 (20–47) a **	0.001
AST, U/L	21 (18–25)	24 (20–28)	21 (16–32)	0.095
ALP, U/L	59 (48–67)	66 (56–85) a **	68 (54–80) a **	0.011
GGT, U/L	15 (12–22)	30 (16–46) a ***	30 (20–51) a ***	<0.001
CRP, mg/L	0.92 (0.50–2.52)	2.30 (1.19–4.50) a ***	3.50 (2.00–6.90) a ***, b **	<0.001
PON1, U/L	308 (194–667)	264 (131–485)	216 (124–413) a *	0.059
AOPP, μmol/L	35.8 (32.9–40.5)	39.4 (32.6–45.9) a **	45.4 (36.2–54.0) a ***	<0.001
TLR9 mRNA	1.1967 (0.9650–1.4296)	1.0743 (0.8368–1.2955)	0.8122 (0.6351–1.0917) a **, b *	0.001
GPX1 mRNA	1.1862 (1.0178–1.4278)	0.9717 (0.6937–1.2560) a *	0.9815 (0.7426–1.2073) a *	0.015
CD36 mRNA	0.8891 (0.7177–1.0553)	0.9823 (0.7436–1.2233)	1.1520 (0.8940–1.2997) a **, b **	0.001
Resistin, ng/mL	23.48 (18.27–27.71)	19.54 (14.98–23.43)	19.07 (14.73–26.30)	0.244
Adiponectin, μg/mL	7.68 (4.90–10.62)	5.49 (3.93–7.82) a *	4.47 (2.53–6.50) a ***, b **	<0.001
Leptin, ng/mL	14.30 (6.09–32.77)	18.47 (8.00–34.40)	31.94 (12.05–46.42)	0.062
AL ratio	0.46 (0.21–1.14)	0.33 (0.15–0.66)	0.15 (0.08–0.37) a ***, b **	<0.001

**Abbreviations:** CG, control group; MASLD, metabolic dysfunction-associated steatotic liver disease; T2D, type 2 diabetes; HbA1c, glycosylated hemoglobin; TC, total cholesterol; TG, triglycerides; HDL-C, high-density lipoprotein cholesterol; LDL-C, low-density lipoprotein cholesterol; ALT, alanine aminotransferase; AST, aspartate aminotransferase; ALP, alkaline phosphatase; GGT, γ-glutamyl transferase; CRP, C-reactive protein; PON1, paraoxonase 1; AOPP, advanced oxidation protein products; mRNA, messenger ribonucleic acid; TLR9, toll-like receptor 9; GPX1, glutathione peroxidase 1; CD36, cluster of differentiation 36; AL, adiponectin-leptin. **Data presentation:** Median (interquartile range) and *p*-value (Quade test). Post hoc: Games-Howell; *p* < 0.05 (*), <0.01 (**), <0.001 (***). Group comparisons: a—MASLD and MASLD + T2D vs. CG; b—MASLD + T2D vs. MASLD.

**Table 3:** Association between risk tertiles and MASLD occurrence using univariate binary logistic regression analysis.

Predictor: MASLD PRESENCE	OR	95% CI	Nagelkerke R^2^	*p*
PON1, U/L *	2.451	1.044–5.754	0.049	0.040
AOPP, μmol/L ***	3.336	1.176–9.462	0.070	0.024
TLR9 mRNA *	1.983	0.770–5.108	0.026	0.156
GPX1 mRNA *	3.132	1.244–7.882	0.073	0.015
CD36 mRNA ***	2.541	1.004–6.432	0.048	0.049
Resistin, ng/mL *	2.689	1.111–6.509	0.057	0.028
Adiponectin, μg/mL *	1.490	0.639–3.474	0.010	0.356
Leptin, ng/mL ***	1.167	0.506–2.690	0.002	0.718

**Abbreviations:** MASLD, metabolic dysfunction-associated steatotic liver disease; OR, odds ratio; CI, confidence interval; PON1, paraoxonase 1; AOPP, advanced oxidation protein products; mRNA, messenger ribonucleic acid; TLR9, toll-like receptor 9; GPX1, glutathione peroxidase 1; CD36, cluster of differentiation 36. **Data presentation:** risk tertile = lowest tertile (*) or highest tertile (***). MASLD presence is a predictor.

**Table 4:** Stepwise multivariate logistic regression models for predictors of high-risk tertiles of CD36 mRNA expression, GPX1 mRNA expression, resistin levels, PON1 levels and AOPP levels in MASLD and CG.

Model	Predictor	OR	95% CI	Nagelkerke R^2^	*p*
Model 1—CD36 mRNA high-risk tertile ***	PON1, U/L	0.997	0.995–1.000	0.119	0.031
Model 2—GPX1 mRNA high-risk tertile *	HDL-C, mmol/L	0.079	0.015–0.420	0.230	0.003
	Resistin	0.992	0.857–0.993		0.031
Model 3—Resistin high-risk tertile *	MASLD presence	2.531	1.041–6.152	0.051	0.040
Model 4—PON1 high-risk tertile *	GPX1 mRNA	0.094	0.022–0.409	0.239	0.002
	CD36 mRNA	11.586	1.897–70.780		0.008
Model 5—AOPP high-risk tertile ***	MASLD presence	5.029	1.548–16.336	0.135	0.007

**Abbreviations:** OR, odds ratio; CI, confidence interval; CD36, cluster of differentiation 36; GPX1, glutathione peroxidase 1; PON1, paraoxonase 1; AOPP, advanced oxidation protein products; HDL-C, high-density lipoprotein cholesterol; MASLD, metabolic dysfunction-associated steatotic liver disease; CRP, C-reactive protein; TLR9, Toll-like receptor 9; GGT, gamma-glutamyl transferase; **Data presentation:** high-risk tertile = lowest tertile (*) or highest tertile (***). **Model 1:** predictors of CD36 mRNA high-risk tertile; adjusted for MASLD presence, age, HDL-C, CRP, PON1 and adiponectin; **Model 2:** predictors of GPX1 mRNA high-risk tertile; adjusted for MASLD presence, age, HDL-C, GGT, PON1 and resistin; **Model 3:** predictors of resistin high-risk tertile; adjusted for MASLD presence, age and GPX1 mRNA; **Model 4:** predictors of PON1 high-risk tertile; adjusted for MASLD presence, age, HDL-C, CD36 mRNA, GPX1 mRNA and adiponectin; **Model 5:** predictors of AOPP high-risk tertile; adjusted for MASLD presence, age, HDL-C, CRP, TLR9 mRNA and adiponectin.

**Table 5:** Association between risk tertiles/quartiles and occurrence of T2D in MASLD using univariate binary logistic regression analysis.

Predictor: PRESENCE OF T2D IN MASLD	OR	95% CI	Nagelkerke R^2^	*p*
PON1, U/L *	1.015	0.513–2.009	<0.001	0.965
AOPP, μmol/L ***	2.897	1.450–5.788	0.085	0.003
TLR9 mRNA *	3.674	1.800–7.497	0.124	<0.001
GPX1 mRNA *	1.224	0.614–2.437	0.003	0.566
CD36 mRNA ***	2.122	1.071–4.202	0.044	0.031
Resistin, ng/mL *	1.422	0.727–2.785	0.010	0.304
Adiponectin, μg/mL *	1.968	0.989–3.917	0.035	0.054
Leptin, ng/mL ***	3.048	1.515–6.132	0.091	0.002

**Abbreviations:** MASLD, metabolic dysfunction-associated steatotic liver disease; T2D, type 2 diabetes; OR, odds ratio; CI, confidence interval; PON1, paraoxonase 1; AOPP, advanced oxidation protein products; mRNA, messenger ribonucleic acid; TLR9, toll-like receptor 9; GPX1, glutathione peroxidase 1; CD36, cluster of differentiation 36. **Data presentation:** risk tertile = lowest tertile (*) or highest tertile (***). MASLD presence is a predictor.

**Table 6:** Stepwise multivariate logistic regression models for predictors of high-risk tertiles of CD36 mRNA expression, TLR9 mRNA expression, leptin levels and AOPP levels in MASLD + T2D and MASLD.

Model	Predictor	OR	95% CI	Nagelkerke R^2^	*p*
Model 1—CD36 mRNA high-risk tertile ***	T2D in MASLD	4.224	1.741–10.246	0.141	0.001
Model 2—TLR9 mRNA high-risk tertile *	T2D in MASLD	3.852	1.612–9.205	0.196	0.002
Model 3—Leptin high-risk tertile ***	T2D in MASLD	2.470	1.171–5.212	0.063	0.018
Model 4—AOPP high-risk tertile ***	TLR9 mRNA	0.240	0.074–0.774	0.085	0.017

**Abbreviations:** OR, odds ratio; CI, confidence interval; CD36, cluster of differentiation 36; TLR9, Toll-like receptor 9; AOPP, advanced oxidation protein products; T2D, type 2 diabetes; MASLD, metabolic dysfunction-associated steatotic liver disease; HDL-C, high-density lipoprotein cholesterol; CRP, C-reactive protein PON1, paraoxonase 1; TG, triglycerides. **Data presentation:** Risk tertile = lowest tertile (*) or highest tertile (***). **Model 1:** predictors of CD36 mRNA high-risk tertile; adjusted for T2D in MASLD, age, HDL-C, CRP, PON1 and adiponectin; **Model 2:** predictors of TLR9 mRNA high-risk tertile; adjusted for T2D in MASLD, age, HDL-C, CRP and AOPP; **Model 3:** predictors of leptin high-risk tertile; adjusted for T2D in MASLD, age, TG and CRP; **Model 4:** predictors of AOPP high-risk tertile; adjusted for T2D in MASLD, age, HDL-C, CRP, TLR9 mRNA and adiponectin.

## Data Availability

The data presented in this study are available on request from the corresponding author. The data are not publicly available due to privacy and ethical considerations.

## Supplementary Materials

The following supporting information can be downloaded at: https://www.mdpi.com/article/10.3390/medsci13040326/s1, Table S1: Primer sequences for qPCR; Table S2: Spearman correlation analysis of CD36 mRNA, GPX1 mRNA, TLR9 mRNA, resistin, leptin, PON1 and AOPP with other continuous markers in all participants; Table S3: Adiponectin, leptin and AL ratio in males (a) and females (b) across study groups.

## Abbreviations

The following abbreviations are used in this manuscript:

MASLD	Metabolic dysfunction-associated steatotic liver disease
T2D	Type 2 diabetes
IR	Insulin resistance
OS	Oxidative stress
MASH	Metabolic dysfunction-associated steatohepatitis
ROS	Reactive oxygen species
AOPP	Advanced oxidation protein products
EMT	Epithelial-to-mesenchymal transition
PON1	Paraoxonase 1
HDL	High-density lipoprotein
LDL	Low-density lipoprotein
PBMCs	Peripheral blood mononuclear cells
CD36	Cluster of differentiation 36
ox	Oxidized
TLR9	Toll-like receptor 9
mt	Mitochondrial
DNA	Deoxyribonucleic acid
GPX1	Glutathione peroxidase 1
CG	Control group
WC	Waist circumference
SBP	Systolic blood pressure
DBP	Diastolic blood pressure
BMI	Body mass index
HCC	Hepatocellular carcinoma
TC	Total cholesterol
TG	Triglycerides
CRP	C-reactive protein
ALT	Alanine aminotransferase
AST	Aspartate aminotransferase
ALP	Alkaline phosphatase
GGT	γ-glutamyl transferase
RNA	Ribonucleic acids
m	Messenger
GAPDH	Glyceraldehyde-3-phosphate dehydrogenase
c	Complementary
AL	Adiponectin-leptin
AHT	Antihypertensives
CVD	Cardiovascular disease
Tx	Therapy
HTN	Hypertension
OAD	Oral antidiabetic drugs
LR	Likelihood ratio
TGF-β	Transforming growth factor β
IL-1β	Interleukin 1β
TNFα	Tumor necrosis factor α
HSC	Hepatic stellate cell
