# Supplementary material for: Transcriptomic, Redox Status and Adipocytokine Profiles in Metabolic Dysfunction-Associated Steatotic Liver Disease: Impact of Coexisting Type 2 Diabetes
Source: Med Sci (Basel). 2025 Dec 18;13(4):326. doi: 10.3390/medsci13040326 (PMC12734822; doi:10.3390/medsci13040326)
Supplement: Supplementary file 1 [file medsci-13-00326-s001.zip › medsci-4024463-supplementary.pdf]

Table S1. Primer sequences for qPCR

Name	Sequence (5'-3')
CD36 forward	AAGTCACTGCGACATGATTAATGG
CD36 reverse	GAACTGCAATACCTGGCTTTTCTC
TLR9 forward	CTGCCACATGACCATCGAG
TLR9 reverse	GGACAGGGATATGAGGGATTTGG
GPX1 forward	CCGGGACTACACCCAGATGA
GPX1 reverse	CACCGTTCACCTCGCACT
GAPDH forward	TGCACCACCAACTGCTTAGC
GAPDH reverse	GGCATGGACTGTGGTCATCAG

Abbreviations: CD36, cluster of differentiation 36; TLR9, toll-like receptor 9; GPX1, glutathione peroxidase 1; GAPDH, glyceraldehyde-3-phosphate dehydrogenase.

Table S2. Spearman correlation analysis of CD36 mRNA, GPX1 mRNA, TLR9 mRNA, resistin, leptin, PON1 and AOPP with other continuous parameters in all participants

Parameter	CD36 mRNA	GPX1 mRNA	TLR9 mRNA	Resistin	Leptin	PON1	AOPP
	ρ	ρ	ρ	ρ	ρ	ρ	ρ
Age, years	-0.112	-0.031	**-0.275*** **	0.034	**0.210** **	0.099	0.057
BMI, kg/m^2	**0.231** **	-0.125	**-0.243** **	-0.134	**0.340*** **	**-0.196** **	**0.204** **
WC, cm	**0.181***	-0.120	**-0.291** **	-0.044	0.114	**-0.227***	**0.249** **
SBP, mmHg	0.073	-0.117	-0.068	-0.018	0.108	**-0.195***	0.120
DBP, mmHg	-0.039	**-0.181***	-0.051	-0.097	0.046	**-0.152***	0.087
Glucose, mmol/L	0.125	-0.073	**-0.392*** **	0.058	**0.165***	-0.108	**0.293*** **
HbA1c, %	**0.228** **	-0.150	**-0.240** **	-0.087	**0.375*** **	**-0.229** **	**0.239** **
TC, mmol/L	**-0.152***	0.011	0.068	-0.060	**0.159***	**0.214** **	0.119
TG, mmol/L	0.087	-0.131	**-0.192***	-0.019	**0.296*** **	-0.137	**0.526*** **
HDL-C, mmol/L	**-0.345*** **	**0.300*** **	**0.299*** **	0.002	-0.007	**0.423*** **	**-0.284*** **
LDL-C, mmol/L	-0.132	0.026	0.126	-0.077	0.121	**0.200***	0.087
ALT, U/L	0.064	-0.144	**-0.151***	-0.083	0.023	**-0.186***	**0.256** **
AST, U/L	-0.051	**-0.171***	-0.040	-0.101	0.002	-0.078	0.127
ALP, U/L	**0.170***	-0.142	-0.060	-0.003	**0.178***	-0.092	0.117
GGT, U/L	**0.155***	**-0.290*** **	**-0.188***	-0.094	0.075	-0.188	**0.221** **
CRP, mg/L	**0.201** **	-0.120	**-0.297*** **	-0.053	**0.349*** **	-0.145	**0.298*** **
CD36 mRNA		0.045	-0.138	0.058	0.117	**-0.228** **	-0.061
GPX1 mRNA	0.045		-0.044	**0.150***	0.132	**0.246** **	0.083
TLR9 mRNA	-0.138	-0.044		-0.114	-0.104	0.122	**-0.295*** **
Resistin, ng/mL	0.058	**0.150***	-0.114		0.044	0.121	0.093
Adiponectin, μg/mL	**-0.222** **	0.076	0.100	-0.058	0.062	**0.264*** **	**-0.230** **
Leptin, ng/mL	0.117	0.132	-0.104	0.044		-0.710	0.071
PON1, U/L	**-0.228** **	**0.246***	0.122	0.121	-0.071		0.001
AOPP, μmol/L	-0.061	0.083	**-0.295*** **	0.093	0.071	0.001	

Abbreviations: mRNA, messenger ribonucleic acid; CD36, cluster of differentiation 36; GPX1, glutathione peroxidase 1; TLR9, toll-like receptor 9; PON1, paraoxonase 1; AOPP, advanced oxidation protein products; BMI, body mass index; WC, waist circumference; SBP, systolic blood pressure; DBP, diastolic blood pressure; HbA1c, glycosylated hemoglobin; TC, total cholesterol; TG, triglycerides; HDL-C, high-density lipoprotein cholesterol; LDL-C, low-density lipoprotein cholesterol; ALT, alanine aminotransferase; AST, aspartate aminotransferase; ALP, alkaline phosphatase; GGT, γ-glutamyl transferase; CRP, C-reactive protein; PON1, paraoxonase 1; AOPP, advanced oxidation protein products; **Data presentation:** ρ - Spearman's correlation coefficient; $p < 0.05$ (*), < 0.01 (**), < 0.001 (***).

Model 1: HDL-C, CRP, PON1 and adiponectin

Model 2: HDL-C, GGT, PON1 and resistin

Model 3: and GPX1 mRNA

Model 4: HDL-C, CD36 mRNA, GPX1 mRNA and adiponectin

Model 5:, HDL-C, CRP, TLR9 mRNA and adiponectin

**Continuous marker (MASLD + CG)**	**HDL-C, mmol/L**	**GPX1 mRNA**	**TLR9 mRNA**	**Resistin**	**Leptin**	**PON1**	**AOPP**
	**ρ**	**ρ**	**ρ**	**ρ**	**ρ**	**ρ**	**ρ**
TC, mmol/L	-0.354***	-0.031	**-0.275*** **	0.034	**0.210** **	0.099	0.057
BMI, kg/m^2	**0.231** **	-0.125	**-0.243** **	-0.134	**0.340*** **	**-0.196** **	**0.204** **
WC, cm	**0.181***	-0.120	**-0.291** **	-0.044	0.114	**-0.227***	**0.249** **
SBP, mmHg	0.073	-0.117	-0.068	-0.018	0.108	**-0.195***	0.120
DBP, mmHg	-0.039	**-0.181***	-0.051	-0.097	0.046	**-0.152***	0.087
Glucose, mmol/L	0.125	-0.073	**-0.392*** **	0.058	**0.165***	-0.108	**0.293*** **
HbA1c, %	**0.228** **	-0.150	**-0.240** **	-0.087	**0.375*** **	**-0.229** **	**0.239** **
	**-0.152***	0.011	0.068	-0.060	**0.159***	**0.214** **	0.119
TG, mmol/L	0.087	-0.131	**-0.192***	-0.019	**0.296*** **	-0.137	**0.526*** **
HDL-C, mmol/L	**-0.345*** **	**0.300*** **	**0.299*** **	0.002	-0.007	**0.423*** **	**-0.284*** **
LDL-C, mmol/L	-0.132	0.026	0.126	-0.077	0.121	**0.200***	0.087
ALT, U/L	0.064	-0.144	**-0.151***	-0.083	0.023	**-0.186***	**0.256** **
AST, U/L	-0.051	**-0.171***	-0.040	-0.101	0.002	-0.078	0.127
ALP, U/L	**0.170***	-0.142	-0.060	-0.003	**0.178***	-0.092	0.117
GGT, U/L	**0.155***	**-0.290*** **	**-0.188***	-0.094	0.075	-0.188	**0.221** **
CRP, mg/L	**0.201** **	-0.120	**-0.297*** **	-0.053	**0.349*** **	-0.145	**0.298*** **
CD36 mRNA		0.045	-0.138	0.058	0.117	**-0.228** **	-0.061
GPX1 mRNA	0.045		-0.044	**0.150***	0.132	**0.246** **	0.083
TLR9 mRNA	-0.138	-0.044		-0.114	-0.104	0.122	**-0.295*** **
Resistin, ng/mL	0.058	**0.150***	-0.114		0.044	0.121	0.093
Adiponectin, µg/mL	**-0.222** **	0.076	0.100	-0.058	0.062	**0.264*** **	**-0.230** **
Leptin, ng/mL	0.117	0.132	-0.104	0.044		-0.710	0.071
PON1, U/L	**-0.228** **	**0.246***	0.122	0.121	-0.071		0.001
AOPP, µmol/L	-0.061	0.083	**-0.295*** **	0.093	0.071	0.001	

Table S3. Adiponectin, leptin and AL ratio in males (a) and females (b) across study groups

Marker	CG – Males	MASLD – Males	MASLD + T2D – Males	*p* (Males)
Adiponectin, μg/mL	5.81 (3.10-8.29)	4.69 (3.54-6.25)	3.39 (2.10-6.36)	0.052
Leptin, ng/mL	5.21 (4.13-6.76)	8.56 (5.49-14.40)	13.11 (9.01-26.68)a**,b*	**0.003**
AL ratio	1.04 (0.48-1.40)	0.46 (0.32-0.90)	0.25 (0.10-0.49)b**	**0.001**
**Marker**	**CG – Females**	**MASLD – Females**	**MASLD + T2D – Females**	***p* (Females)**
Adiponectin, μg/mL	8.44 (5.90-13.16)	5.95 (4.87-10.02)	5.19 (3.63-7.01)a**,b*	**0.004**
Leptin, ng/mL	19.17 (12.06-34.83)	30.46 (19.20-46.05)	40.41 (25.34-61.29)	0.065
AL ratio	0.31 (0.18-0.83)	0.20 (0.14-0.38)	0.13 (0.07-0.28)a**,b*	**0.003**

Abbreviations: AL, adiponectin-leptin; CG, control group; MASLD, metabolic dysfunction-associated steatotic liver disease; T2D, type 2 diabetes. **Data presentation:** Median (interquartile range) and p-value (Quade test). Post hoc: Games-Howell; p < 0.05 (*), < 0.01 (**), < 0.001 (***). Group comparisons: a – MASLD and MASLD + T2D vs CG; b – MASLD + T2D vs MASLD.
